# A serological study of canine herpesvirus-1 infection in a population of breeding bitches in Norway

**DOI:** 10.1186/1751-0147-56-19

**Published:** 2014-04-02

**Authors:** Anette Krogenæs, Vibeke Rootwelt, Stig Larsen, Lena Renström, Wenche Farstad, Arve Lund

**Affiliations:** 1 Department of Production Animal Clinical Sciences, Faculty of Veterinary Medicine and Biosciences, Norwegian University of Life Sciences, Postboks 5003, NO-1432 Ås, Norway; 2 Department of Companion Animal Clinical Sciences, Faculty of Veterinary Medicine and Biosciences, Norwegian University of Life Sciences, Postboks 5003, NO-1432 Ås, Norway; 3 Center of Epidemiology and Biostatistics, Faculty of Veterinary Medicine and Biosciences, Norwegian University of Life Sciences, Postboks 5003, NO-1432 Ås, Norway; 4 Department of Virology, Immunobiology and Parasitology, National Veterinary Institute, Ulls Väg 2B, 751 89 Uppsala, Sweden; 5 Department of Health Surveillance, Norwegian Veterinary Institute, Ullevålsveien 68, Postboks 750 Sentrum, N-0106 Oslo, Norway

**Keywords:** Canine herpesvirus, Dog, Sero-epidemiology, Reproduction

## Abstract

**Background:**

Canine herpesvirus-1 (CHV1) causes a fatal hemorrhagic disease in neonatal puppies and is associated with infertility in female dogs. This study was conducted to assess the status of CHV1 infection in bitches in proestrus or estrus and to investigate possible risk factors by a detailed questionnaire. Blood samples were collected from healthy bitches (n = 193) not vaccinated against CHV1, aged one year or older and admitted for estrus control to the Canine Reproductive Clinical Unit, Norwegian School of Veterinary Science. The serum samples were analysed by immunoperoxidase monolayer assay and serum titers were recorded as the reciprocal value of the highest dilution producing specific cell staining.

**Results:**

Altogether, 85.5% of the dogs had CHV1 titers ≥ 80 and were classified as positive. Mean age for dogs included in the study was 4.2 years (95% CI 4.0-4.5), and there was no difference in age between seronegative dogs *vs* seropositive dogs. When grouping the seropositive dogs into three categories according to the magnitude of the titer, a total of 38.8% of the bitches displayed a weakly positive titer of 80, 44.8% had moderately positive titers of 160 or 320 and 16.4% of the dogs fell into the strongly positive category with titer of ≥640. No association was demonstrated when comparing CHV1 antibody titers to fertility parameters such as previous matings, pregnancies, whelpings, puppies born or condition of puppies. Further, there was no difference in seroprevalence between bitches that had been abroad for a period of time and dogs only living within a Norwegian environment*.* Samples from dogs collected in summer and fall displayed moderate to high antibody titers indicating recent infection with CHV1. Season, previous birth, and participation in competitions/shows explained 67-78% of the variation in antibody titer.

**Conclusions:**

This study demonstrates that CHV1 infection is common in breeding bitches in the eastern part of Norway. Associations with putative risk factors were not identified. However, season, previous whelping, and participation in competitions/shows explained 67-78% of the variation in antibody titer.

## Background

There has been an increasing concern among dog breeders in Norway about canine herpesvirus-1 (CHV1) and its ability to cause reproductive problems in the bitch and perinatal puppy loss [1]. Several studies report high seroprevalence in the dog population in many European countries and there is increased movement of dogs between Norway and continental Europe.

CHV1 is an alphaherpesvirus which was first reported from the USA in the early 1960s [2]. In neonatal puppies, the virus can produce a systemic fatal haemorrhagic infection causing focal necrosis in parenchymatous organs of puppies up to 2-weeks-old [3,4]. CHV1 is of low pathogenicity in puppies older than 5 weeks [5]. In the adult dog, the infection is usually asymptomatic, but may cause upper respiratory infection [3] and ocular disease [6,7]. However, in the bitch herpesvirus infection has been associated with reproductive problems, such as low conception rate, fetal resorption, abortion, stillborn or weak puppies and small litter size both in experimental [8,9] and epidemiological studies [10-12]. Oronasal transmission is considered to be the main route of infection, but genital and transplacental transmission may also occur [8,13,14].

In a recent study from Norway, 80% of the dogs in the general adult population were classified as seropositive [15]. No difference was observed between genders, but a significant geographical variation was demonstrated. In other countries the reported prevalence of antibodies against CHV1 varies from approximately 20% to 94% in healthy dog populations [16-18]. Some studies have reported the median antibody titer to be significantly higher in dogs from kennels with reproductive problems than in those from kennels without fertility disorders [10,19]. Different factors might influence the seroprevalence in populations such as age distribution, number of dogs in kennels and households and hygienic conditions, but the conclusions have been inconsistent [11,12,15,19,20].

The aim of this study was to estimate the seroprevalence of CHV1 antibodies in reproductively active bitches and to investigate possible risk factors.

## Material and methods

### Study design, animals and sampling

A cross-sectional study design was applied in this investigation. A total of 193 purebred bitches admitted for routine estrus control to the Canine Reproductive Clinical Unit at the Norwegian School of Veterinary Science were included in the study. All were healthy, one year of age or older (up to 9-years-old) and had not been vaccinated against herpesvirus infection. The dogs came from eastern Norway. All dog breeds were accepted for sampling and 66 different breeds are represented in this material. Of the 193 blood samples, 89 samples were collected during winter (December–February), 74 during spring (March–May), 20 during summer (June–August) and 10 during fall (September–November). In total, 57.3% of the bitches had been pregnant earlier, whereas the remaining came for control before their first mating.

The study was conducted in agreement with the provisions enforced by the Norwegian Animal Research Authority (NARA).

The dog owners supplied information according to a questionnaire including a number of putative risk factors as last estrus, previous mating, whelping and the status of puppies. Further, information about travelling abroad, participation in dog shows, competitions and hunting trials the last year were included (see Additional file 1).

The dog owners received information on the background and purpose of the study and gave their written consent.

In the clinic, the routine procedure included ocular inspection of vulva, examination of vaginal smears to evaluate cells from vagina and progesterone analysis of serum to estimate ovulation time and optimal time for mating or artificial insemination. In addition, blood samples were taken for analysis of antibodies against CHV1. These blood samples were allowed to clot at room temperature for 1–3 hours before centrifugation at 3000 rpm for 10 minutes. The serum fraction was carefully harvested and kept at −20°C until analysis.

### Immunoperoxidase monolayer assay (IPMA)

The IPMA for demonstrating antibodies to CHV1 was performed as described [15] and previously used both in epidemiological [10,15] and prospective [21] studies. IPMA is a virus neutralisation test. Briefly, monolayers of CHV infected Madin-Darby canine kidney cells in 96-well microtiter plates were incubated with twofold dilutions 1:10 to 1:1280 of test sera. Following washing and incubation steps, secondary peroxidase-conjugated rabbit anti-dog immunoglobulin G was added (DAKO, Denmark), and subsequently the substrate 3-amino-9-ethylcarbazole (Invitrogen AB, Sweden). Positive and negative serum samples were included. Serum titers were recorded as the reciprocal value of the highest dilution producing specific cell staining. Titers equal to or above 80 were considered positive for exposure to CHV1. Further, increasing antibody titers were categorized as; 80 = weakly positive, 160 and 320 = moderately positive, 640 ≥ strongly positive.

The validation of the IPMA showed a sensitivity of about 90% compared to an in-house ELISA test (National Veterinary Institute, Sweden) used in a vaccine trial on sera from dogs vaccinated against CHV. Twenty-seven serum samples were collected from 13 dogs at intervals during the trial. Estimation of the specificity is not available.

### Statistical analyses

Categorical variables are given in simple contingency tables with numbers and percentages. Continuously distributed variables are expressed as mean values with 95% confidence intervals. All comparisons between groups were performed two-tailed and differences considered significant at a level of 5%.

Contingency Table Analysis was first performed for comparison of groups with regard to categorical variables [22]. For comparison of assumed continuously distributed variables, Analysis of Variance (ANOVA) was used [23]. The variables which in the single analysis indicated differences between positive and negative titers were included in a forward and backward stepwise logistic regression model [24]. The CHV1 titer categories were used as the dependant variables. The set of independent variables contributing to discriminate against negative titer were season, previous birth and participation in competitions. This set was included in the final logistic regression analysis. Reciever Operating Characteristics (ROC) analysis was used to estimate the accuracy of CHV1 titer classification. The accuracy is said to be good if the area under the ROC-curve (Auc) is larger than 0.7. An area close to 0.5 represents no difference against negative titer. The logistic model was evaluated using the Hosmer-Lemeshow test and ROC analysis [25].

## Results

No pathological conditions were found during the clinical examination and none of the dogs received any kind of medical treatment at that time. During the last year, only one dog had been treated to end a previous estrus and none of the dogs had received long-lasting cortisone treatment. Altogether 85.5% of the dogs had CHV1 antibody titers ≥ 80 and were classified as positive. Mean age for dogs included in the study was 4.2 years (95% CI 4.0-4.5), and there was no difference in age between the groups of seronegative dogs *vs* seropositive dogs. Further, there were no significant differences in titer categories between dogs aged one, two and up to nine years old. No association was demonstrated when comparing CHV1 antibody titers and fertility parameters such as previous matings, pregnancies or whelpings. Furthermore, no difference was observed between negative and positive dogs with respect to information on the condition of puppies born previously (Table 1).

**Table 1:** **Variables within positive *vs *** **negative titer categories**

**Variables**	**Outcome**	**Titer category**		**p-values**
		**Negative**	**Positive**	
**Previous pregnancy**	No	10	72	
	Yes	18	92	0.61
	Missing	0	1	
**Previous whelping**	No	14	83	
	Yes	14	80	0.73
	Missing	0	2	
**Condition of puppies born previously**	Normal	10	60	
	Weak	0	1	0.83
	Some dead	4	18	
	Missing	0	3	
**Previous unsuccessful attempt to get pregnant**	No	21	122	
	Yes	7	40	0.62
	Missing	0	3	
**Stay abroad last year**	No	10	70	
	Yes	18	90	0.33
	Missing	0	5	
**Participation in competitions last year**	No	0	18	
	Yes	28	141	0.017
	Missing	0	6	

All of the bitches with negative titers had participated in dog shows and competitions, whereas 11.3% of the positive bitches had never attended such activities (*P* = 0.017). There was no association between antibody titer and travel abroad (Table 1).

**Figure 1:**
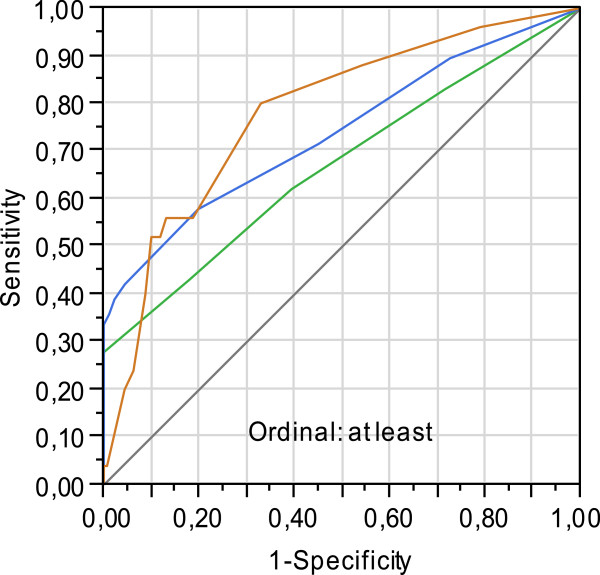
**Receiver Operating Characteristic (ROC) including season, previous birth and competitions/shows.** Weakly positive (green line) results in an area of 0.67, moderately positive (blue line) 0.74 and strongly positive (orange line) 0.78. The accuracy is said to be good if the area under the ROCcurve (Auc) is larger than 0.7. An area close to 0.5 represents no difference against negative titer.

Of all positive dogs, 38.8% displayed a titer of 80, whereas 44.8% and 16.4% of the dogs fell into the moderately and strongly positive categories, respectively (Table 2). There was no difference in age between the different titer categories. Further, there were no significant differences between the different titer categories with respect to previous matings, pregnancies and whelpings or conditions of puppies born. However, though not significant different, 11 of 27 (40.7%) dog owners in the highest titer category reported problems with getting the bitches pregnant after mating compared to 7 of 28 (25.0%) of the negative dogs and 29 of 135 (21.5%, 3 missing observations) in the weakly and moderately positive titer categories (Table 2). Travel abroad had no influence on titer value, whereas significantly fewer dogs within all positive titer categories had participated in dog shows, competitions or hunting trials compared to dogs with negative titers (*P* = 0.01, Table 2). Samples from all the negative dogs were collected during winter and spring. Of the positive dogs, 81.8% (n = 135) samples were collected during these two seasons. During summer and fall, only bitches from the two highest titer categories were represented (*P* < 0.01, Table 3). By multivariable analysis, it was shown that each of the parameters season, previous whelping, and participation in competitions/shows contributed significantly (67-78%) to the different antibody titer categories (Figure 1).

**Table 2:** **Variables within the three positive *vs *** **negative titer categories**

	**Outcome**	**Titer categories**				**p-values**
		**Negative**	**Weakly**^**a **^**positive**	**Moderately**^**b **^**positive**	**Strongly**^**c **^**positive**	
**Previous pregnancy**	No	10	33	29	10	
	Yes	18	31	44	17	0.53
	Missing	0	0	1	0	
**Previous whelping**	No	14	37	33	13	
	Yes	14	26	40	14	0.68
	Missing	0	1	1	0	
**Condition puppies born previously**	Normal	10	20	30	10	
	Weak	0	1	0	0	0.78
	Some dead	4	5	10	3	
	Missing	0	0	0	1	
**Previous unsuccessful attempt to get pregnant**	No	21	51	55	16	
	Yes	7	12	17	11	0.34
	Missing	0	1	2	0	
**Stay abroad last year**	No	10	28	30	12	
	Yes	18	36	41	13	0.24
	Missing	0	0	3	2	
**Participation in competitions last year**	No	0	5	11	2	
	Yes	28	59	59	23	0.01
	Missing	0	0	4	2	

^a^Weakly positive = 80.

^b^Moderately positive = 160 and 320.

^c^Strongly positive ≥ 640.

**Table 3:** Canine herpesvirus-1 serological classification in the total material (n = 193) by season of the year

**Titer**	**Winter**^**a**^	**Spring**^**b**^	**Summer**^**c**^	**Fall**^**d**^	**Total**
	**No (%)**	**No (%)**	**No (%)**	**No (%)**	**No (%)**
**Negative**	17 (8.8)	11 (5.7)	0 (0)	0 (0)	28 (14.5)
**Weakly positive**	37 (19.2)	27 (14.0)	0 (0)	0 (0)	64 (33.2)
**Moderately positive**	32 (16.6)	25 (13.0)	11 (5.7)	6 (3.1)	74 (38.3)
**Strongly positive**	3 (1.6)	11 (5.7)	9 (4.7)	4 (2.1)	27 (14.0)
**Total**	89 (46.1)	74 (38.3)	20 (10.4)	10 (5.2)	193 (100)

^a^Winter: December-February.

^b^Spring: March-May.

^c^Summer: June-August.

^d^Fall: September-November.

## Discussion

This study shows that the majority of clinically healthy bitches presented to the Canine Reproductive Clinical Unit at the Norwegian School of Veterinary Science, have been infected by CHV1.

In a previous Norwegian study including both genders, we demonstrated significant geographical differences ranging between 58.5% in eastern Norway and 98% in mid-Norway [15]. When comparing the seroprevalence in the two dog populations originating from eastern Norway and collected in the same period, there is a difference of 85.5% (breeding bitches in this study) *vs* 58.5% (dogs of both genders in the previous study). This finding might indicate that reproductively active bitches admitted to the reproduction clinic have a greater risk of CHV1 exposure and subsequent infection compared to the general dog population in this part of the country.

All bitches in the present study were in proestrus or estrus. Although clear evidence of an association between circulating reproductive hormone concentrations and viral activation have not been demonstrated, Ronsse *et al.*[12] reported fluctuations in CHV1 antibody titers related to cycle stage. They found slightly higher titers in estrus and significantly lower titers in early di-estrus [11]. Both 17-β estradiol and medroxyprogesterone acetate have been shown to promote herpes simplex virus type 1 reactivation in mice [26,27]. Evermann *et al.*[20] listed risk factors for reproductive disease in the bitch, and cycle stage was identified as one of 8 factors having a positive correlation with disease. Whether estrus itself is a potential stressor which can reactivate a latent infection or increase susceptibility for a new infection remains to be seen. However, clinical signs of reactivation have been described to occur more frequently during heat and around parturition [14]. Psychological stress has also been suggested to contribute to reactivation of latent herpes simplex virus in humans [28,29].

In Norway, few breeding dogs are kept in kennels, so our data are not directly comparable to kenneled dogs in studies from other countries. Babaei *et al.*[30] and Ronsse *et al.*[18] could not demonstrate differences in CHV1 seroprevalence between privately owned in- house pets and kennelled dogs. Nöthling *et al.*[19] observed that seroprevalence was independent of kennel size, whereas Ronsse *et al.*[11] found higher titers in kennels with 6–30 dogs than in those with fewer dogs.

There was no difference in age between the seronegative and the seropositive bitches, and the seroprevalence did not increase with age. This is in contrast to our previous study [15] and can be explained by less variation in age in the breeding bitches in this study compared to dogs in the general population.

In the current study, there were no significant correlations between titer category and breeding status, such as previous matings, previous whelping, failing to get the bitch pregnant or conditions of puppies. This is in accordance with Ronsse *et al.*[18]. In the present study, the time between previous mating and sampling is variable, and the titer at previous mating is unknown. But, interestingly, owners reported increasing problems of getting the bitches pregnant after previous mating in the highest titer category compared to the others. Recent CHV1 infection coinciding with early stage of pregnancy may result in fetal loss and this could be a plausible explanation. Several authors have demonstrated an association between CHV1 serological status and reproductive problems. Dahlbom *et al.*[10] found that dogs from kennels with reproductive problems had significantly higher CHV1 titers than dogs from kennels without reproduction problems. Ronsse *et al.*[11] demonstrated an association between serological status and a history of abortion in bitches. Furthermore, Van Gucht *et al.*[31] found a relation between the presence of positive breeding bitches and neonatal death and/or infertility in the kennel.

Travel abroad with breeding dogs is quite common in Norway and is confirmed by dog-owners in this investigation for both antibody positive and –negative dogs. However, travel abroad had no influence on the titer values. This was also the fact when this was tested as a separate risk factor in our previous study of dogs in different parts of Norway [15]. By multivariable analysis, it was shown that the variable travel abroad contributed to a better classification of seropositive dogs in our previous study, which was not the case in this study with fewer dogs included. Surprisingly, significantly fewer dogs within all positive titer categories had participated in dog shows, competitions or hunting trials compared to dogs with negative titers. This might be related to the fact that the owners of the bitches in this study are committed, well-informed breeders and even though they travel or participate in different competitions, they keep their dogs under more controlled conditions than dog owners in general. The attitude and behavior of the owners in addition to the very low number of dogs not participating in competitions, might therefore be possible confounders in this study. We can conclude that the endemic status of CHV1 infection in the Norwegian dog population provides ample opportunities for spread of the virus. Staying abroad or participation in competitions/shows does not seem to add an extra risk of contracting infection in this study.

In our data, 57.3% of the dogs had been pregnant prior to sampling. No difference in seroprevalence between this group of bitches and those being mated for the first time was demonstrated, indicating that the oronasal infection route is the most likely way of virus transmission between dogs. This finding is in accordance with other studies [12,15,18] of adult dog populations. However, Babaei *et al.*[30], suggest that oronasal transmission of CHV1 may be epidemiologically less important than venereal transmission. In their study, no seropositive dog was detected in animals younger than 12 months of age. It might be important to emphasize that the overall CHV1 seroprevalence was estimated to 20.7%, which is very low.

In spite of the high seroprevalence in reproductively active dogs, we have the opinion that fertility problems and loss of newborn puppies due to CHV1 infection at present are of minor importance in Norway. There are no epidemiological data available regarding the prevalence of reproduction disorders in bitches. However, two recent studies on postnatal puppy mortality have been published. IndrebØ *et al.*[32] reported a total puppy loss to be 6.9% during the first three weeks of life in a selected population of four breeds. Etiological diagnosis was not recorded, and the authors concluded that impact of CHV1 infection was unlikely because of rare occurrence and no evidence indicating this disease. TØnnessen *et al.*[33] reported perinatal mortality in a large-scale observational study including 10.810 litters and 224 breeds. In total, perinatal mortality was observed in 8% of the puppies (4.3% stillborn and 3.7% died before age of 8 days). Autopsy was not performed and causes for perinatal mortality not pursued. Obviously, there is need for follow-up investigations to find etiological causes of these clinical manifestations. At the Norwegian Veterinary Institute in Oslo, CHV1 has been diagnosed sporadically in newborn puppies (Øyvor KolbjØrnsen, personal communication). There is most likely an underdiagnosing of CHV1 infection because few puppies are submitted for autopsy due to high costs. In Norway, dogs are kept in households usually including one family dog, occasionally two, and there are few big breeding kennels [34]. Management and hygienic conditions are generally good and the stress level low probably contributing to less impact of CHV1. The fact that most bitches are seropositive to CHV1 following natural immunization, may protect the puppies against disease. Further, satisfactory vaccine coverage against other diseases, provide fetuses and puppies with good general protection against infection.

Interestingly, moderately positive and highly positive bitches were found only in dogs sampled during summer and fall, whereas no negative or weakly positive dogs were sampled in these seasons. This might indicate that the dogs are more exposed to new infections or re-expression of latent infections during the summer and fall, which could be due to increased outdoor activities in general and contact with other dogs in this period. Our findings may explain observations by TØnnessen *et al.*[33] that litters born during fall had a higher risk of experiencing early neonatal mortality than litters born during other seasons.

The three parameters of season, previous birth and participation in competitions/shows increase the probability of correct classification between the different titer categories to between 70 to 80%*.*

Vaccination against CHV1 infection is used in some countries. In Norway, the herpesvirus vaccine is a non-core vaccine. Vaccination is not routinely recommended for breeding bitches. Nevertheless, veterinarians experience an increased demand for vaccination from owners concerned about CHV1 infection and impact of their breeding success. In case of an increased risk of CHV1 infection by recent contact with other bitches that have aborted, borne weak puppies or suffered increased neonatal mortality due to CHV1, vaccination should be considered in young females entering into their first or second pregnancy. In bitches with a positive CHV1 antibody titer from natural infection, protective efficacy is difficult to evaluate. We consider the status of majority of these dogs as being protected. Moreover, if reactivation of virus occurs, this is likely to boost the immune response and increase level of immunity. However, there might be relevant to consider vaccination even though naturally acquired antibodies are present, for example in older breeding bitches from about six years age. Here a reactivation may be more likely to cause problems since aging is associated with decreased immune responsiveness and increased susceptibility to infectious diseases [20,35,36].

## Conclusions

This study demonstrates that CHV1 infection is common in reproductively active bitches. There is no correlation between high seroprevalences and previously reported reproductive disorders in connection to matings, pregnancies, whelpings or mortality in newborn puppies in the bitches’ life. Travelling abroad the last year did not influence antibody titer. All of the negative bitches, but only 81.8% of the positive bitches had participated in competitions/shows the last year, which indicates that in this data, these activities were not related to increased risk of herpesvirus infection. By multivariable analysis, it was shown that each of the parameters season, previous birth, and participation in competitions/shows explained 67-78% of the variation in antibody titer.

## Abbreviations

CHV1: Canine herpesvirus-1; IPMA: Immunoperoxidase monolayer assay; ANOVA: Analysis of variance; ROC: Reciever operating characteristics; Auc: Area under the ROC-curve.

## Competing interests

The authors declare that they have no competing interests.

## Authors’ contributions

AK, AL, VR and SL participated in the discussion on the study design. AK did the clinical work, collected the samples and performed the questionnaires. LR was responsible for the serum analysis. AK, AL, VR, SL and WF participated in interpretation of the data. AK, AL, VR, SL, WF and LR helped to draft the manuscript. AK wrote the final manuscript. All authors read and approved the final manuscript.

## Supplementary Material

Additional file 1: Questionnaire used in the study. Click here for file
