# Supplementary material for: A serological study of canine herpesvirus-1 infection in a population of breeding bitches in Norway
Source: Acta Vet Scand. 2014 Apr 2;56(1):19. doi: 10.1186/1751-0147-56-19 (PMC4021736; doi:10.1186/1751-0147-56-19)
Supplement: Additional file 1: — Questionnaire used in the study. [file 1751-0147-56-19-S1.docx]

**Additional file 1**

**Questionnaire used in the study**

**Questionnaire**

**Dog identification:**

Date:

Owner’s name:

Address:

Mobile phone:

Breed:

Born:

Vaccinated against CHV1:

**The following questions have 3 alternative answers:**

**Mated earlier:** No/ yes/ do not know

**Born puppies:** No/ yes/do not know

**Condition of puppies at birth:** Dead/weak/normal

**Previous attempt to get pregnant without success:** No/yes/do not know

**Disease or medical use now:** No/yes/do not know If yes: Which disease/medicaments?

**Medical control of reproduction last year:** No/yes/do not know If yes: Which medicament?

**Long-lasting cortisone treatment last year:** No/yes/do not know

**Travel abroad last year:** No/yes/do not know If yes: Which country?

**Participation in competitions/shows last year:** No/yes/do not know
